# ‘A bite before bed’: exposure to malaria vectors outside the times of net use in the highlands of western Kenya

**DOI:** 10.1186/s12936-015-0766-4

**Published:** 2015-06-25

**Authors:** Mary K Cooke, Sam C Kahindi, Robin M Oriango, Chrispin Owaga, Elizabeth Ayoma, Danspaid Mabuka, Dennis Nyangau, Lucy Abel, Elizabeth Atieno, Stephen Awuor, Chris Drakeley, Jonathan Cox, Jennifer Stevenson

**Affiliations:** Faculty of Infectious and Tropical Diseases, London School of Hygiene and Tropical Medicine, London, UK; Kenya Medical Research Institute Centre for Global Health Research/Centers for Disease Control and Prevention, Kisumu, Kenya; Johns Hopkins Malaria Research Institute, Johns Hopkins Bloomberg School of Public Health/Macha Research Trust, Choma, Zambia

**Keywords:** Malaria, Exophagic, Endophagic, *Anopheles funestus*, *Anopheles arabiensis*, LLIN, IRS, Kenya, Highlands

## Abstract

**Background:**

The human population in the highlands of Nyanza Province, western Kenya, is subject to sporadic epidemics of *Plasmodium falciparum*. Indoor residual spraying (IRS) and long-lasting insecticide treated nets (LLINs) are used widely in this area. These interventions are most effective when *Anopheles* rest and feed indoors and when biting occurs at times when individuals use LLINs. It is therefore important to test the current assumption of vector feeding preferences, and late night feeding times, in order to estimate the extent to which LLINs protect the inhabitants from vector bites.

**Methods:**

Mosquito collections were made for six consecutive nights each month between June 2011 and May 2012. CDC light-traps were set next to occupied LLINs inside and outside randomly selected houses and emptied hourly. The net usage of residents, their hours of house entry and exit and times of sleeping were recorded and the individual hourly exposure to vectors indoors and outdoors was calculated. Using these data, the true protective efficacy of nets (P*), for this population was estimated, and compared between genders, age groups and from month to month.

**Results:**

Primary vector species (*Anopheles funestus* s.l. and *Anopheles arabiensis*) were more likely to feed indoors but the secondary vector *Anopheles coustani* demonstrated exophagic behaviour (p < 0.05). A rise in vector biting activity was recorded at 19:30 outdoors and 18:30 indoors. Individuals using LLINs experienced a moderate reduction in their overall exposure to malaria vectors from 1.3 to 0.47 bites per night. The P* for the population over the study period was calculated as 51% and varied significantly with age and season (p < 0.01).

**Conclusions:**

In the present study, LLINs offered the local population partial protection against malaria vector bites. It is likely that P* would be estimated to be greater if the overall suppression of the local vector population due to widespread community net use could be taken into account. However, the overlap of early biting habit of vectors and human activity in this region indicates that additional methods of vector control are required to limit transmission. Regular surveillance of both vector behaviour and domestic human-behaviour patterns would assist the planning of future control interventions in this region.

**Electronic supplementary material:**

The online version of this article (doi:10.1186/s12936-015-0766-4) contains supplementary material, which is available to authorized users.

## Background

The feeding locations and the biting times of individual *Anopheles* spp. could potentially confound assessments of their role in local malaria transmission [1, 2]. There is evidence that in Kenya and elsewhere in Africa, primary vectors and other potentially important secondary malaria vectors do not feed exclusively within houses [1, 3–14] and that significant levels of vector exophagy, feeding outdoors, can occur at times when the human population is still outdoors [5, 7, 11–13, 15, 16]. Malaria eradication has recently returned to the global health agenda for the first time since the failure of the Global Malaria Eradication Programme (GMEP) of the 1950s and 1960s [17–20]. The development of insecticide resistance, and the exophily and exophagy of *Anopheles* species (resting and feeding outdoors) are thought to be among the key contributors to the failure of the original p rogramme [21] which relied heavily on indoor residual spraying (IRS) with DDT. It has, therefore, been suggested that any future campaign to achieve eradication, still less elimination, may fail if the lessons learnt from the collapse of the GMEP are forgotten or ignored [20, 22].

Today, vector malaria elimination plans are heavily reliant on the use of long-lasting insecticide treated nets (LLINs) and IRS, both of these being strategies that are theoretically less effective against the malaria vectors that are fully or partially exophilic or exophagic [23]. Successful malaria control is threatened by the emergence of physiological, biochemical or behavioural adaptations within the vector population in response to the use of insecticide [24, 25]. IRS and LLINs require direct contact between the mosquito and surfaces carrying sufficient levels of insecticide to kill or repel the vector. Pre-existing or adapted feeding and resting behaviour may reduce or negate this contact [19].

The feeding behaviour and circadian rhythms of *Anopheles* are genetically determined [26, 27], with the former being linked with inversion polymorphisms [26]. There is an added complication of intraspecies variation, where mosquitoes of the same species but different homokaryotypes react to identical environmental conditions in different ways [26]. There has been some debate surrounding the importance of pre-existing exophilic and exophagic *Anopheles* populations when planning control efforts [1, 19, 28–30]. Whilst the occurrence and mechanisms of insecticide resistance over the last century have been well documented in African *Anopheles* populations [21, 25, 31], the extent to which the emergence of population-wide vector behavioural change in response to control methods, known as ‘behaviouristic resistance’, affects the use of nets and IRS remains unclear. This can only be established by observing vector population behaviour in the field and there is a lack of basic pre-intervention baseline studies [12, 25, 31–34].

The time of feeding in both endophagic and exophagic populations may also be of critical importance if it occurs in the hours outside of LLIN use [16, 28, 30, 35–38], particularly in areas where nets are the main control intervention used [1].There have been reports of net and IRS use leading to a reduction in indoor biting or resting, and a shift to exophagic behaviour, earlier feeding times or feeding on different hosts [10, 39–48]. In Kenya, a pronounced reduction in endophily was observed in the vectors *Anopheles gambiae* sensu stricto (s.s.) and *Anopheles funestus* sensu lato (s.l.) and a shift in host preference from humans to other mammals after 5 years of bed-net use [44]. Similarly, host choice change in *An. funestus* s.l. was observed by Githeko et al. following use of permethrin-impregnated eave-sisal curtains [49]. In Benin, *An. funestus* s.l. populations exhibited increased exophagy and a shift in feeding times after LLIN introduction and demonstrated a shift to diurnal feeding in a recent study in Senegal [50, 51]. For these species complexes, this could be due to a change of the sibling species composition, rather than a behavioural change of a single species *per se*, as some members demonstrate higher zoophagy and exophagy than others. This was demonstrated in Kenya where following mass net distribution the *An. gambiae* s.s. population decreased and the remaining sibling species *Anopheles arabiensis,* demonstrated higher exophagy and zoophagy [52]. In Tanzania, substantial reduction in the indoor resting and a small increase in the exophagic behaviour of *An. gambiae* s.s. was recorded after the introduction of pyrethroid-impregnated bed nets in one study village [39]. It should also be noted, that these changes are not universal, a recent study in Kenya noted that late night vector feeding behaviour still persisted in areas 10 years after bed net distribution [53].

Human behaviour may also influence the extent of human-vector contact. Entomological studies carried out in Zambia and Tanzania incorporated the proportion of the human population indoors but not asleep and those indoors and asleep under an LLIN, in order to calculate the protective efficacy of bed nets [37, 38, 45]. The methodology of these studies provides a useful insight into the true protective efficacy of bed nets when both human and vector behaviours are combined but are partially limited, as they do not estimate the area-wide effects on the vector population that universal coverage of LLIN can offer [54].

The World Health Organization recommends that adequate baseline information is collected in an area before residual insecticide is used [55]. Without a good understanding of the baseline entomological situation, the emergence of true behavioural adaptations will be difficult to detect. This concern has led to a call for regular monitoring of vector feeding behaviour as control programmes are expanded [37]. Regrettably, as noted by Smits et al., vector control is susceptible to a reduction in supervision and evaluation when activities have been in place for some time [4]. Success is more likely if control efforts are designed to adapt to changing local conditions [4]. Without a baseline vector dataset it is difficult to identify the emergence of behaviouristic resistance, and the accuracy of malaria transmission models used to plan future control efforts will be compromised [56–58].

This study aimed to assess the behaviour of exophagic or partially exophagic malaria vectors in Rachuonyo South, western Kenyan highlands, over different seasons, and to assess the level of exposure to *Anopheles* bites that individuals experience when not protected by an LLIN. Using vector exposure calculations, the protective efficacy of nets was calculated for this population.

## Methods

### Study site

The current Kenyan national malaria strategic plan aims to reduce morbidity and mortality caused by malaria, using current control tools, including regular national mass distributions of LLINs and IRS in selected regions [59]. The western Kenyan highlands are considered an area of unstable *Plasmodium falciparum* transmission and prone to epidemics, and as such are included in those areas selected for intensive malaria control by universal LLIN distribution and either annual or intermittent IRS [60–62]. Malaria transmission in this region is characterized by marked temporal and spatial heterogeneity [49, 63, 64]. The identification of malaria vectors, their behaviour and the contribution of each vector to local transmission are key to evaluating the success of control measures, and to planning future campaigns [2, 37, 56, 57]. This is particularly important in areas of unstable transmission which constitute key targets for eliminating the disease as vector dynamics can vary dramatically by season [65–67]. In Nyanza Province, western Kenya, a number of descriptive studies have been carried out in Kisii district of vector distribution and behaviours in the context of control interventions [68, 69]. However in the highland fringe area of neighbouring Rachuonyo South, a district of approximately 200,000 population bordering the highly endemic lake area, no recent data exist on vector bionomics.

This study was carried out under the highland Malaria Transmission Consortium in southern Nyanza Province, Kenya in the adjacent villages of Lwanda and Siany, in Rachuonyo South District (0°25′59.53″ S, 34°55′40.36″ E; altitude 1,420–1,570 m ASL). This location was previously identified as an area of relatively high *P. falciparum* transmission during cross-sectional and cohort parasitological surveys carried out in 2009 and 2010 and with indoor-resting anopheline populations [70]. IRS had been carried out by the local health services in this region in 2010 using Fendona (alphacypermethrin), a year before the study began, and was repeated in July 2011 using Icon (Lambda-cyhalothrin). This area was also included in the mass distribution of LLINs during the rainy season (April–June) in 2011, as part of the Kenyan National Malaria Strategy [71]. However, prior to the distribution in 2011, 100% of the 48 houses recruited into the present study already owned a minimum of one net (and more than half of the households owned two or more nets).

In western Kenya the primary vectors of *P. falciparum* are considered to be*, An. arabiensis,An. funestus* and *An. gambiae* s.s., three of the six malaria vector species identified in Kenya [72, 73]. There is some evidence that the once widely distributed *An. gambiae* s.s. has declined in recent years and that *An. arabiensis* has encroached upon its previous distribution [52, 73, 74]. This shift has been attributed to the wide-scale use of insecticide-treated nets (ITNs) [44, 52].

### Sample size

The study was designed to compare the catch of light-traps set outdoors with those placed indoors over 1,800 trap nights, 900 trap nights for each study arm over a 1-year period. To test the null hypothesis that there was no difference between the mean density of primary malaria vector species feeding inside and outside houses, data from a previous field study in the region were used to estimate minimum sample sizes. As there was the potential for intracluster correlation caused by repeated sampling at trap locations, formulae for community studies from Hayes and Bennett were used [75]. The minimum sample size required to compare *An. gambiae* s.l. feeding inside and outside, with 80% power, 95% precision and a coefficient of variation of 0.8 was 7.9 traps in each study arm per night, giving a total of 16 traps in use per study night. Using the same power, precision and coefficient estimates, a total of 8.4 traps per study arm would be required to compare the mean catches of *An. funestus* s.l. As previous studies had been disrupted by unexpected weather conditions (outdoor catches, in particular, can be interrupted by heavy rain), a conservative total of 24 traps, 12 indoors and 12 outdoors, running each night was selected for the study.

### Mosquito collection

Fieldwork was carried out between February 2011 and May 2012. Community sensitization, recruitment, mapping and a pilot study took place between February and May 2011. Sampling began in June 2011 and continued for six nights every lunar month (with the exception of December 2011) until the end of May 2012, a total of 75 collection nights. Sampling was scheduled on nights near a new moon to minimize the effect of moonlight on the outdoor light-trap collection and to reduce bias when comparing species distribution and flight activity across seasons [76–78]. An estimate of the presence and period of moonlight was calculated using a lunar calendar and the method described by Bowden [77, 79].

A stratified random sampling method was adopted to minimize sampling bias when selecting sampling locations and to reduce variance in the dataset [80]. The study site was identified with the aid of satellite imagery (Quickbird Inc, Longmont, CO, USA), with a spatial resolution of <1 m, which could therefore be used to identify structures on the ground. Using GIS software (ArcGIS 9.2, Redmond CA, USA), a sampling grid was defined to divide the area into 36 quadrants (300 m × 300 m) covering an area of 1.8 sq km running across the valley floor and a portion of the adjoining hillsides.

**Figure 1:**
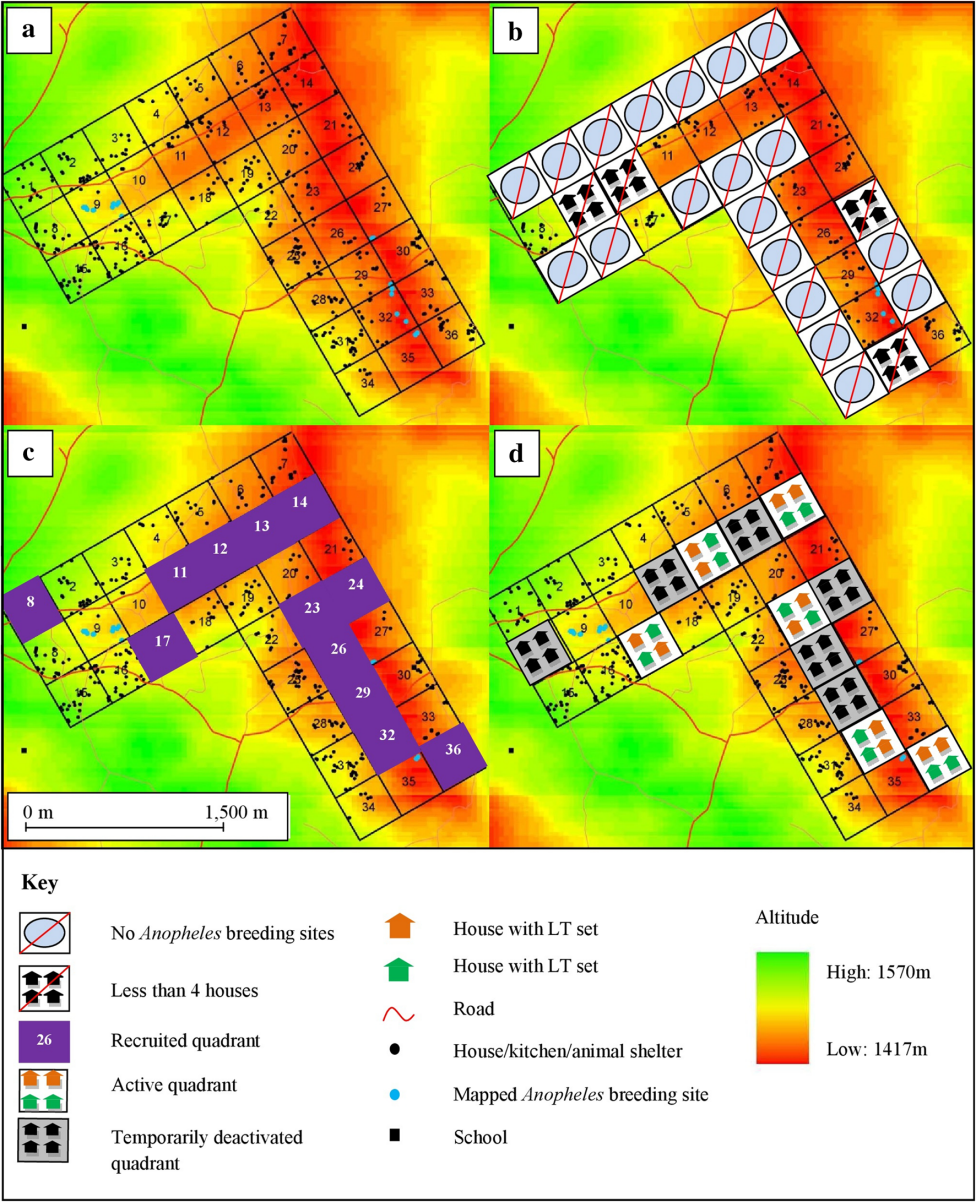
Maps of the study site showing the sampling quadrants, and phases of recruitment. **a** Construction of sampling grid and identification of building structures using aerial maps; **b** Survey of sampling grid to identify and exclude quadrants without breeding sites or with fewer than four houses; **c** Randomization of houses within the remaining quadrants and sequential recruitment of four houses per quadrant; **d** An example of a typical night of sampling, with six quadrants active and six quadrants deactivated.

A survey of the selected quadrants was conducted on the ground. Quadrants with permanent breeding sites (n = 13) were selected for recruitment, as these have been associated with higher adult vector productivity in highland areas than temporary breeding sites, and are more likely to be present throughout the sampling year [81] (Figure 1). Quadrants with fewer than four occupied houses were omitted from the recruitment. Remaining eligible quadrants were randomized and processed sequentially until 12 quadrants had been recruited into the study. Within each quadrant the mapped houses were randomized and four households with associated light-trap workers were recruited into the study. During recruitment, data on house construction, occupant numbers, bed nets, local IRS activity, and domestic animal ownership were collected.

To reduce selection bias six quadrants (i.e., 24 houses) were randomly selected for trapping each night. Within quadrants, two houses were randomly selected for outdoor sampling with the remaining two allocated for indoor trapping. As the effective range of light-traps has been estimated at 5 m [82], outdoor sampling took place at least 10 m from the house to reduce the chance of the inhabitants acting as unshielded bait. A miniature CDC light-trap with a standard 6.3 V incandescent bulb (Model 512, John W Hock, Florida, USA), with an LLIN occupied by a light-trap worker, was used to trap mosquitoes. Traps set indoors were hung in the sleeping quarters and traps set outside were hung adjacent to an occupied temporary, open-sided rain shelter constructed from a domed one-man tent (Kenya Canvas, Nairobi, Kenya).

Traps were checked and connected by 17:30 and the light-trap worker replaced the collection cups every hour until 22:30. The traps inside the houses continued to run until 05:30 the next morning when the collection cup was changed for the final hour. For traps set outside, no collections were made between 22:30 and 05:29 as it was assumed that all residents would be indoors between these times. These times were based on a survey of sleeping times carried out by Battle, recording sleeping times in rural Nyanza province, and are consistent with previous assumptions on sleeping times in rural areas and the scope of *Anopheles* activity [1, 83]. Between 05:29 and 06:30, a final hour of trapping was carried out both inside and outside. Supervisors made random checks throughout the night, every night, to ensure traps were running and set up correctly.

Mosquitoes were killed by freezing, and morphologically identified to genus and species level using morphological keys [84, 85]. A subsample of female *Anopheles* that were neither blood fed, gravid nor semigravid were dissected for determination of parity status as a proxy for age [86]. Samples were stored in 0.5-ml micro centrifuge tubes packed with silica gel crystals and transported to the Centre for Global Health Research, Kenya Medical Research Institute/Centers for Disease Control and Prevention in Kisian, Kisumu (CGHR, KEMRI/CDC), for further analysis. Sibling species of the *An. gambiae* complex were identified using an *An. gambiae* specific diagnostic PCR [87]. The presence of *P. falciparum* or *Plasmodium vivax* CSP in specimens was tested by ELISA using an established methodology used by CGHR, KEMRI/CDC, adapted from techniques described by Beier et al. and Wirtz et al. [88, 89].

### Population sleeping and behaviour survey

Questionnaires were used to gather information on the time people entered and exited their houses in the evening and morning, the time they slept and their use of bed nets. The head of each household used a digital watch to complete the questionnaire on behalf of all adults and children that slept in that house. Questionnaires were distributed and completed twice during each six-night sampling period, on a week night and a Saturday night, and collected the next day. Questionnaires were not distributed during the sampling week in December 2011 due to the short study period.

### Statistical analysis

The location and times of *Anopheles* feeding behaviour were analysed using a random-effects negative binomial model accounting for repeated measurements using Stata (Version 11, StataCorp LP, Texas, USA). Bivariate analysis was carried out to assess the role of potential confounders, not on the causal pathway, against the outcome of interest. Those variables deemed not significant (p > 0.05) were discarded. Independent variables were then tested for correlation using a Pearson’s product-moment correlation test. Those demonstrating multicollinearity (correlation > 0.90) were identified and one variable, from the two tested, chosen for the model. In all analyses, a predetermined significance level of p < 0.05 for the incident rate ratio (IRR) was sufficient evidence that the null hypothesis could be rejected. A model was deemed a poor fit if the Wald Chi squared test statistic (***χ***^***2***^) had a p > 0.05.

To determine whether there were groups within the local human population that were at greater risk of exposure to malaria vectors than others, the mean catch of *An. funestus* s.l., *An. arabiensis* and *An. gambiae* s.s. trapped by hour and location were extracted for each sampling week and the man biting rate (MBR) for each hour that the traps were running was calculated for both locations. The potential exposure of individuals to these vectors was then estimated using each individual’s responses to the sleeping questionnaire for the sampling week that the questionnaire was completed, thus creating a dataset that reflected any change to the vector-human interaction throughout the sampling year.

Human exposure to malaria vectors and the true protective efficacy of bed nets was calculated using methods adapted from the work described by Geissbuhler et al., based on the formulae published by Killeen et al. [37, 45] (see Additional file 1). These earlier studies calculated the protective efficacy of bed nets as a result of reduced exposure to *An. gambiae* bites, incorporating the proportion of the population indoors but not asleep and those indoors and asleep under an ITN. In the present study, calculations were made for exposure to the three primary vectors *An. funestus* s.l., *An. arabiensis* and *An. gambiae* s.s.

In this region it is rare for individuals to sleep outdoors at night, and this was excluded from the analysis. A limitation of this method is the necessary assumption that the protective efficacy of the bed nets (P) is uniform between houses, and that each individual used an identical model and age of bed net, and used it correctly. There was a mass distribution of LLINs during this study, but there was evidence of older LLINs in use within the recruited households. In this calculation the functional protective efficacy of LLINs is assumed to be 80% (P = 0.8), which had been adopted by previous studies informed by existing evidence from experimental hut trails [37, 45]. We have also reported estimates that assume functional protective efficacy to be 100% for comparison purposes with other studies. Pairwise Kruskal–Wallis (K–W) analysis was used to compare P* between participant age groups and month of data collection.

### Ethics

Informed consent was obtained from those participating in the study. This work was reviewed and approved by the KEMRI/National Ethics Review Committee, Kenya (SSC No. 2007) and by the Ethics Committee of the London School of Hygiene and Tropical Medicine, UK. Informed consent was obtained from the head of each household recruited into the study and from every light-trap worker.

## Results

### *Anopheles* species identification and feeding behaviour

A total of 3,330 *Anopheles* were trapped between June 2011 and May 2012. Based on morphological identifications, the greatest proportion of female *Anopheles* were the vector species *An. funestus* s.l. (n = 1,475, 44%) and *An. gambiae* s.l. (n = 263 8%). *Anopheles funestus* s.l. was the species most frequently trapped both inside and outside houses (inside: n = 1,099, 69% of females caught, and outside: n = 376, 33%). A total of 2,750 (99%) of all *Anopheles* trapped were examined using *An. gambiae*-specific diagnostic PCR to identify sibling species. The remaining 1% of samples examined did not contain sufficient material to analyse. Using PCR, 145 were identified as *An. arabiensis* (inside: n = 110, and outside: n = 35) and five samples were confirmed as *An. gambiae* s.s. (inside: n = 5, and outside: n = 0). The remainder did not amplify when tested, the majority of which had been morphologically identified as *An. funestus* s.l. Due to logistical constraints, PCR was not carried out to identify members of the *An. funestus* complex. This is a recognized limitation of this study which should be addressed by ongoing studies to genetically sequence these specimens.

**Figure 2:**
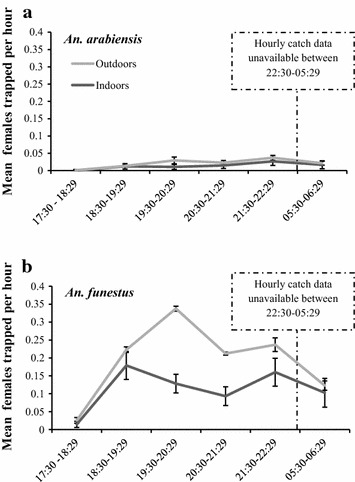
Mean hourly catch of **a** *Anopheles arabiensis* and **b** *Anopheles funestus* s.l. caught by CDC light-traps. Traps were emptied hourly between 17:30 and 22:29 each evening and between 05:30 and 06:29 the next morning.

When comparing indoor and outdoor catches directly at times when traps were running concurrently, there was evidence that *An. funestus* s.l. were more likely to feed indoors than outdoors (IRR = 1.5, 95% CI: 1.1-2.010, p = 0.006) (Table 1). This species complex was also more likely to be trapped indoors when carrying eggs, when either semigravid or gravid (IRR = 4.5, 95% CI 2.5–8.2, p < 0.005). Combined, a total of 18.9% (n = 174) *An. funestus* s.l. were identified as either semi-gravid or gravid. For collections carried out between the hours of 17:30 and 22:29 and 05:30 and 06:30 when people are likely to be outside of a net, *An. funestus* s.l. biting increased indoors between 18:30 and 19:29 ($\overline{x}$ = 0.18, 95% CI 0.14–0.22) and 19:30 and 20:29 ($\overline{x}$ = 0.13, 95% CI 0.10–0.15) with a third rise between 21:30 and 22:29 ($\overline{x}$ = 0.16, 95% CI 0.12–0.20) (Figure 2). However, there was no evidence to indicate that the numbers recorded for these hours differed significantly (p > 0.1). When compared directly to the numbers caught between 21:30 and 22:29, fewer *An. funestus* s.l. were likely to be trapped indoors very early in the evening (17:30–18:29: p < 0.001), between 20:30 and 21:39 (p = 0.020) and in the early morning, 05:30–06:29 (p < 0.001). Outdoors *An. funestus* s.l. females fed later between 19:30 and 20:29 (*x* = 0.21, 95% CI 0.13–0.22) carrying through to 21:30–22:29 (*x* = 0.076, 95% CI 0.06–0.096, p < 0.001).

**Table 1:** Female *Anopheles* morphologically identified vector species between the hours of 17:30 and 22:29 and 05:30 and 06:30

Outcome measure	Total number *Anopheles* caught by trap location		Comparison between indoors and outdoors with outdoor IRR = 1		
	Indoor	Outdoor	Indoor IRR (95% CI)	P	Wald *χ* ^*2*^ (p)
Primary African malaria vector species					
*An. funestus* s.l.	544	376	1.5 (1.1–2.010)	0.006	18 (<0.001)
*An. arabiensis*	67	35	1.9 (1.03–3.4)	0.038	17 (0.0023)
*An. gambiae* s.s.	4	0	NC	NC	NC
*An. nili*	1	1	NC	NC	NC
Other documented Kenyan *Anopheles* species					
*An. coustani*	19	151	0.15 (0.090–0.25)	<0.001	64 (<0.001)
*An. demeilloni*	63	148	0.42 (0.26–0.68)	<0.001	37 (< 0.001)
*An. dthali*	2	4	0.52 (0.080–3.3)	0.49	2.3 (0.32)
*An. gibbinsi*	1	11	0.13 (0.015–1.08)	0.059	3.6 (0.059)
*An. longipalpis*	2	5	NC	NC	NC
*An. maculipalpis*	17	55	0.31 (0.16–0.58)	<0.001	25 (<0.001)
*An. natalensis*	1	3	NC	NC	NC
*An. parensis*	1	2	NC	NC	NC
*An. pretoriensis*	9	29	0.41(0.18–0.94)	0.035	9.02 (0.011)
*An. rufipes*	5	26	0.204 (0.078–0.54)	0.001	13 (0.0012)
*An. salbaii*	2	7	NC	NC	NC
*An. squamosus*	3	21	0.22 (0.056–0.86)	0.029	9.6 (0.0081)
*An. symesi*	4	4	1.03 (0.22–4.7)	0.97	0.00 (0.97)
*An. ziemanni*	1	1	NC	NC	NC

*NC* negative binomial statistical model could not converge.

*Anopheles arabiensis* was also caught in both indoor (n = 67) and outdoor traps (n = 35) and, was also more likely to feed indoors (IRR = 1.9, 95% CI 1.03–3.4, p = 0.038) (Table 1). A total of 12.7% (n = 13) *An. arabiensis* were identified as either semi-gravid or gravid. Indoor *An. arabiensis* biting activity started in the early evening between 18:30 and 19:29 ($\overline{x}$ = 0.012, 95% CI 0.0042–0.020) and 19:30 and 20:29 ($\overline{x}$ = 0.011, 95% CI 0.0033–0.018) with a second rise in MBR between 21:30 and 22:29 ($\overline{x}$ = 0.026, 95% CI 0.015–0.040) (Figure 2). However, there was no evidence to indicate that the two periods of increased activity differed in intensity (p > 0.1). Outdoor biting started later in the evening with activity increasing between 19:30 and 20:29 ($\overline{x}$ = 0.019, 95% CI 0.01–0028) and continuing until 22:29 (p < 0.001). There was significantly less activity in the early hours of the evening (18:30–19:29: p < 0.05) when compared to the numbers recorded between 21:30 and 22:29.

A total of four *An. gambiae* s.s. females were trapped between the hours of 17:30 and 22:29 and 05:30 and 06:29, all indoors. The increase in the indoor mean hourly MBR occurred between 20:30 and 21:29 ($\overline{x}$ = 0.0027, 95% CI −0.0010 to 0.0064). There were insufficient data to make a comparison between the hour of biting or the numbers of *An. gambiae* s.s. found inside and outside.

A smaller number of samples were morphologically identified as those that have been previously documented in Kenya and may represent infrequent or secondary malaria vectors [14, 73, 90]. Of these, *An. coustani*, *Anopheles demeilloni*, *An. maculipalpis*, *Anopheles pretoriensis, Anopheles squamosus,* and *Anopheles rufipes* females were predominantly trapped outdoors (p < 0.05). Samples of other species were too few in number to fit the model (Table 1).

There was evidence that older *Anopheles* females that had previously laid eggs (parous mosquitoes) were more likely to bite outdoors (p < 0.05) and, conversely, that younger nulliparous females were more likely to feed indoors (p < 0.05). However, when analysing the catch of malaria vector species: *An. funestus* s.l. (55% parous indoor, 78% outdoor)*, An. arabiensis* (78% indoor, 80% outdoor) and *An. gambiae* s.s. (100% indoor, 0% outdoor) there was either insufficient data to fit a model, or the model did not fit well (Wald χ^2^ p > 0.05). There was a similar difficulty when fitting models to the other *Anopheles* species that had been dissected (Wald χ^2^ p > 0.05), with the exception of *An. coustani.* A total of 44 *An. coustani* were successfully dissected, with 77% (n = 34) identified as parous (indoor n = 4, 12% and outdoor n = 30, 88%). There was some evidence that parous *An. coustani* females were more likely to forage outdoors (IRR = 0.26, 95% CI 0.091–0.77, p = 0.05).

### Entomological inoculation rate (EIR)

A subset (n = 2,706, 98%) of female *Anopheles* were tested for the presence of *P. falciparum* and *P. vivax* CSP, these samples included those from indoor traps left running between 22:30 and 05:30. Five samples were not tested due to sample damage. Of the samples tested, *P. falciparum* CSP was detected in 44 samples (1.6%) (Table 2). The majority of infected *Anopheles* were morphologically identified as *An. funestus* s.l. (n = 30, 69%, 2.0% CSP positive). Other morphologically identified species included *An. demeilloni* (2.7% CSP positive) *An. gibbinsi* (7.7% CSP positive) and *An. longipalpis* (12.5% CSP positive). One sample of An. *arabiensis* (contained *P. falciparum* CSP (0.7%). *Plasmodium vivax* CSP was not detected from any of the samples tested.

**Table 2:** Percentage of *P. falciparum* CSP positive, blood fed and parous primary vector species trapped between the hours of 17:30 and 22:29 and 05:30 and 06:30

Primary vector species	% CSP positive		% blood fed		% parous	
*An. funestus* s.l.	2.0%	(n = 30)	14.1%	(n = 130)	66%	(n = 126)
*An. arabiensis*	0.7%	(n = 1)	13.7%	(n = 14)	79%	(n = 11)
*An. gambiae* s.s.	0.0%	(n = 0)	0.0%	(n = 0)	100%	(n = 1)
*An. nili*	0.0%	(n = 0)	50%	(n = 1)	0.0%	(n = 0)

The estimated annual EIR was calculated using the indoor collections, as indoor data spanned the complete sampling night from 17:30 to 05:30 the next morning. The EIR for this region, was 20 (95% CI 17–22) *P. falciparum*-infected bites per person per year. Estimates of the mean indoor EIR per person per night were calculated for the study period and these ranged between no infected bites per person per night and a maximum of 0.27 (95% CI 0.22–0.32) recorded in March 2012.

### Protective efficacy of bed nets

**Figure 3:**
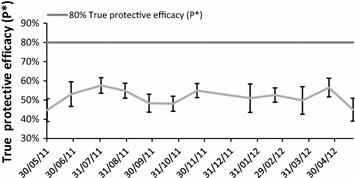
Monthly mean true protective efficacy of nets (P*) against the combined bites of primary malaria vectors. For the purpose of this study, primary malaria vectors are defined as *An. nili, An. funestus* s.l. and *An. gambiae* s.l.

The true mean bed net protective efficacy (P*), calculated as efficacy against the combined bites of primary malaria vectors (see Additional file 1) was estimated at 51% (95% CI 50–53%) if nets were assumed to offer protection against 80% of vector bites and 64% (95% CI 62–66%) if they were 100% effective. This equates to a drop in efficacy of 29% (95% CI 27–30%) if bed nets are assumed to offer protection against 80% of vector bites when used correctly. The P* calculated for each sampling month ranged from 45 to 56% (Figure 3). Protective efficacy varied significantly across the sampling year when taking into consideration the protection offered against the bites of all primary malaria vectors (K–W χ^2^ = 37, 11 df, p = 0.0001), *An. funestus* s.l. alone (K–W χ^2^ = 37, 11 df, p = 0.0001), *An. arabiensis* (K–W χ^2^ = 230, 11 df, p = 0.0001) and *An. gambiae* s.s. (K–W χ^2^ = 170, 11 df, p = 0.0001).

The estimated proportion of indoor and outdoor exposure to malaria vectors fluctuated significantly across the sampling year (K–W χ^2^ = 147, 11 df, p = 0.0001) (Figure 3), with a peak in the proportion of outdoor exposure to the primary vectors in early October 2011 (with bed net: 27%, 95% CI 19–34% and without bed net: 9.7%, 95% CI 7–12%). When tested using the two-sample Mann–Whitney test, there was no significant difference in the outdoor exposure to malaria vectors between men and women (M–W, z = 0.35, p = 0.72), or between participants’ exposure on a week night as opposed to a night at the weekend (M–W, z = 1.1, p = 0.26).

**Figure 4:**
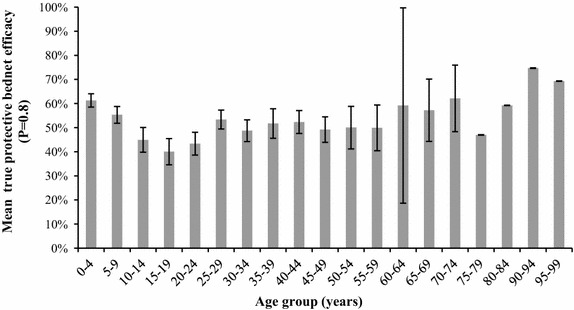
Variation in mean true protective efficacy of nets (P*) by age group of participants. Calculations based on a bed net efficacy where nets are estimated to prevent 80% of bites when used correctly.

The P* of LLINs also varied with the age group of participants (K–W χ^2^ = 147, 18 df, p = 0.0001), for *An. funestus* s.l. alone (K–W χ^2^ = 144, 18 df, p = 0.0001), *An. arabiensis* (K–W χ^2^ = 119, 17 df, p = 0.0001) but it was not significant for the small number of *An. gambiae* s.s. trapped (K–W χ^2^ = 14, 13 df, p > 0.1). When individual age groups were compared against the reference age group of under 9 years, those aged 10–59 had significantly different levels of P* than those aged under 9 years (p < 0.001), and examination of the medians and means indicate that the levels of P* are lower in these age groups (Figure 4).

### Indoor versus outdoor exposure

**Figure 5:**
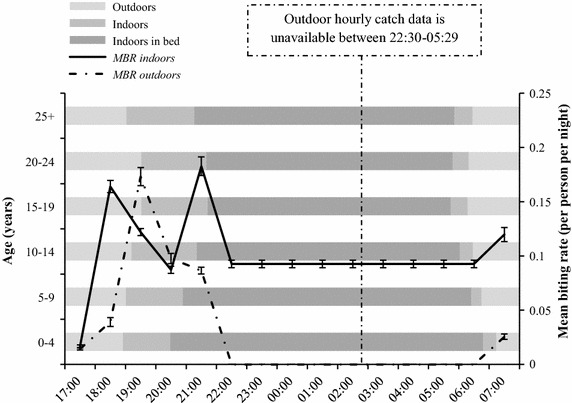
Combined hourly man biting rate (MBR) for *Anopheles arabiensis* and *Anopheles funestus* s.l. Biting activity overlaid on the reported movements of the local human population indoors and outdoors before, during and after sleep (mean hours). Data for outdoor hourly MBRs were not collected between the hours of 22:30–05:29. For diagrammatic proposes, data for indoor MBR estimates between the hours of 22:30–05:29 were divided equally across the 7 h of collection. Data collected between June 2011 and May 2012.

Based on the times recorded during the survey, it was estimated that individuals not using bed nets would experience a mean of 95% of their total vector exposure inside their houses (95% CI 95–96%), and 5% outdoors (95% CI 4–5%). It was estimated that a mean 31% (95% CI 29–33%) of their daily exposure occurred indoors before they went to bed. A mean of 64% (62–66%) of daily exposure occurred while they were asleep. When individuals used bed nets their estimated mean exposure reduced from 1.3 vector bites per night (95% CI 1.2–1.3%) to 0.47 (95% CI 0.44–0.51) (Figure 5).

## Discussion

In common with the previous work carried out in Zambia and Tanzania to determine the protective bed net efficacy, this study highlights the importance of integrating human behaviour into the assessment of human-vector contact in relation to malaria transmission [16, 37, 38, 45]. Despite predominantly endophagic primary vectors in this region, the overall P* was low at 51% (95% CI 50–53%) and this may be explained by exposure occurring indoors at times of the evening before nets are used which equates to 31% of total mean daily exposure. This is substantially lower than the bed net efficacy using similar methods reported from rural Tanzania [37], but higher than that reported from urban Tanzania where *An. arabiensis* is predominantly exophagic [45]. In the present study, 90–95% of vector exposure was calculated to occur within the house if LLINs were not used, which is similar to levels reported for *An. funestus* s.l. in Zambia [38] and the results of a study of matched surveys of human and mosquito behaviour from Burkina Faso, Tanzania, Zambia, and Kenya [91]. The use of LLINs in the present study reduced an individual’s exposure from 1.3 bites per night to 0.47 bites per night. In agreement with a recent study carried out in Western Kenya the majority of exposure occurred indoors [53], an estimated 65% of mean daily exposure occurred during sleeping hours, indicating that nets still may offer personal protection in an area of low transmission.

The two primary vector species *An. funestus* s.l. and *An. arabiensis* were both active inside and outside from 18:30 onwards, two-and-a-half hours before the mean time local residents reported going to bed. When studying mosquito activity outside times when individuals are likely to be asleep, the peak hours of biting varied between species, but universally very little activity occurred during the early evening (17:30–18:29) and morning (05:30–06:29). The latter may be due to the low dawn temperatures in this area, but the former may have been influenced by the heat and light intensity in the hours before dusk. During the times studied, *An. funestus* s.l. demonstrated a distinct bimodal pattern of indoor feeding activity, with the first increase in biting activity between 18:30 and 20:30 followed by a second at 21:30 and 22:29. Although there was no evidence that these periods differed in intensity (p < 0.05), they were both significantly higher than the preceding or interim hours (p < 0.05).

The residents of this area reported that 90% used nets, greater than that previously recorded in Kakamega in the western Kenyan highlands (56%) [92], or by the Malaria Indicator Survey in 2010, 61% [62]. However, the former survey was conducted in a different area with a different ethnic populations. Furthermore, the area of the current study was a research site where active health teams had been working for the past 2 years and data were collected during a year of mass LLIN distribution with prolonged marketing campaigns to increase awareness and adherence. Net use recorded in the present study may not reflect wider patterns of bed net use.

It is important to note that this study, in common with previous work [16, 37, 38, 45], did not estimate the area-wide effects on the vector population that may result from universal coverage of LLIN [54]. It has been shown that mass distribution will reduce transmission of principally endophagic vectors by reducing the reservoir of disease [16]. The P* estimated here may be an underestimation as it does not include any potential community-wide effects.

*Anopheles funestus* s.l. was the most abundant primary vector species trapped in the area throughout the year with an indoor MBR of 0.15–1.2 and an outdoor MBR of 0.13-1.2 bites per person per night. Similar findings were reported from lowland areas in Nyanza Province [93]. *Anopheles funestus* s.s. is considered the anthropophagic exception in a complex of zoophagic species [94], so it is likely that the *An. funestus* s.l. in this study contain other morphologically identical members of the complex. Work continues to genetically sequence the full set of anophelines caught to confirm species identities. Alternatively, it is possible that the LLIN and IRS use in this area has induced this species to seek alternative hosts. Such phenotypic, plastic feeding behaviour has been observed in *An. gambiae* s.s., which can demonstrate zoophilic behaviour in field conditions if their preferred human hosts are not readily available [95]. This shift from anthropophagy to zoophagy was noted in Kenyan *An. funestus* s.l. populations in response to permethrin-impregnated eaves-sisal curtains [42] but again no data were given as to the sibling species of the complex.

*Anopheles arabiensis* was also present in the study site, with a peak MBR of 0.12 bites per person per night. This is not consistent with either the historical distribution of this species or recent work carried out in the Nandi hills, where *An. gambiae* s.s. females were more prolific than *An. arabiensis* [72, 96]. However, these findings do align with the observations of Ndenga et al. who surveyed larval breeding sites above 1,500 m in neighbouring Western province, where *An. arabiensis* represented a third of the *An. gambiae* s.l. larvae collected [74]. *Anopheles arabiensis* is found at high densities in lowland Nyanza and it is therefore conceivable that this species has encroached upon the neighbouring highland fringe areas, filling the niche left by *An. gambiae* s.s., which was selectively targeted by local control efforts [41, 44, 52, 68]. It is possible that the distribution of *An. arabiensis* may have always included highland areas, with this species being overlooked by those studies that predominantly used indoor traps that do not target outdoor-resting and feeding species [74].

EIR estimates were higher than those previously reported for similar areas of western Kenya [49, 63]. Ndenga et al. reported an EIR of 0.2–1.1 in highland areas of the neighbouring district Kisii Central and in Kakamega (neighbouring province) and Githeko et al. recorded a peak EIR of 12.8 from comparable elevations in Kakamega [49, 63]. Those studies may have underestimated the EIR as they used pyrethrum spray catches, which will not trap endophagic and exophilic *Anopheles* that are infected but exit the house early. Furthermore, in the current study, the site was specifically selected due to high *P. falciparum* prevalence and incidence and high indoor-resting densities of anopheline mosquitoes. Within this area of higher transmission, only houses within quadrants that contained breeding sites were selected, and thus the EIR from the present study could be interpreted as that of a transmission ‘hotspot’ [97]. In common with studies that used methods other than human landing catches (HLC) to estimate EIR [98], the present study did not include an estimation of outdoor transmission and thus potentially overestimated the total exposure an individual will experience throughout the year. In addition to these limitations, it is also possible that the EIR may be overestimated. This study did not include steps to limit false-positive CSP-ELISA results by reanalysing the homogenate therefore it is possible that false-positives were included in the EIR estimate [99].

Across all *Anopheles* species trapped, there was evidence (p < 0.05) that females carrying eggs were 4.5 times more likely to feed indoors, potentially presenting a higher transmission risk indoors as these mosquitoes are older than nulliparous females. However, unfed parous females without eggs are used as a proxy for older females and were more likely to bite outdoors (p < 0.05) and, conversely, younger nulliparous females were more likely to feed indoors (p < 0.05). Therefore, the number of gravid females caught in traps indoors may reflect the recruitment of the female indoor-resting population that are attracted to the CDC-light trap during egg development.

The findings of this study support the hypothesis that the levels of both LLIN and IRS coverage are currently not sufficient to interrupt transmission in this setting. IRS should be an effective control tool in a region where the majority of exposure occurs inside the house and should complement the use of LLINs if biting occurs before times of net use and/or the observed exophagy is also accompanied by indoor-resting behaviour. IRS was and is still implemented in Rachuonyo district but coverage at the time was not universal, with 38% of houses sprayed in the previous 12 months [62]. Improving the coverage of the current IRS campaign may be more effective, but if conducted poorly it may also encourage the development of insecticide resistance. Therefore, as the majority of exposure is currently occurring indoors, measures to bar entry to *Anopheles* may be a cost-effective option to complement existing interventions. These could include the use of ceilings, window and door screens, measures that have successfully reduced the number of *Anopheles* indoors both historically and in experimental hut trials [100, 101].

An important limitation of the present study is the use of light-traps outdoors. Light-traps have been in use since the early part of the 20th century, and have been used widely in a variety of transmission settings, including Africa [56, 82, 102]. These traps work on the principle that the mosquito is drawn into the ‘dazzle zone’, at which point the fan mechanism sucks them into the trap [78, 102]. The exact mechanics of this process and the extent to which it is species-specific are not well understood [102, 103]. The type and size of catch may be influenced by a number of factors, including the species of mosquito [78], the model of trap and the wavelength of the light used [102] and whether the strength of illumination can be kept constant. Indeed, it is reasonable to assume that the traps used during the present study could not achieve a uniform level of illumination throughout the night.

Light-traps have several practical advantages: they are commercially available which aids standardisation [104], they are easily accepted by communities within study sites [105] and they have low running costs. A number of experiments have been carried out to establish whether light-trap catches correlate well with those from HLC and some studies have indicated that light-trap catches of *Anopheles* have relatively high sporozoite rates [103–105]. Other studies have reported no significant difference between sporozoite rates from light-traps and HLC, with a corresponding similarity in parity rates between these trapping methods [106–108]. With a lack of standardisation between studies, there appears to be no definitive evidence to indicate whether light-traps, with or without human bait, can catch the anthropophagic vector population.

It has been claimed that CDC light-traps cannot be used outdoors [109], yet this appears to be based on limited evidence. The small number of studies that assessed HLC with light-traps hung outside tended to place the light-traps directly under the eaves of houses [110, 111], either with an accompanying light-trap inside the same house [110, 112] or with no accompanying human bait [110, 113]. Costantini et al. (1998) did hang CDC light-traps away from houses, under a thatched rain shelter with human bait, but found no correlation between its catch and that of HLC when comparing *An. gambiae* s.l. However, when *An. funestus* numbers were compared there was a density-dependent correlation between the catch of the outdoor HLC and the CDC light-trap [114]. The authors concluded that outdoor traps were not effective but acknowledged that this was based on a limited data set [114]. Overgaard et al. (2012) used a CDC light-trap with a UV bulb outdoors but with no human bait and reported a correlation between the numbers of *An. gambiae* s.l. and *An. melas* trapped by the two light-traps. The authors did, however, express some doubts about the practicality of using light-traps outdoors with such low numbers and such high levels of variability between catches [110]. Currently, there is insufficient evidence to definitively dismiss the use of light-traps outdoors as a means of collecting anthropophagic *Anopheles*. Where HLC is not available, light-traps remain one of the few viable trapping methodologies not designed solely to catch the resting *Anopheles* population, and may represent a useful tool to catch the vector population.

The present study contributes to the knowledge of both primary and secondary vector species dynamics in the fringe area of the western Kenyan highlands. The existence of predominantly exophagic potential secondary vector species such as *An. coustani* and *An. demeilloni* should be an important consideration when planning future control efforts, as they are likely to be overlooked during campaigns targeted at the primary vector species that feed indoors during sleeping hours. These species have the potential to maintain low levels of transmission in this area. It is therefore vital that entomological surveillance should be carried out on a regular basis in this area and in other regions of unstable malaria transmission targeted for malaria control or future malaria elimination.

## Conclusions

The present study indicates that primary vectors are more likely to feed indoors in the fringe of the western Kenyan highlands. Exophagic behaviour does occur, but when considered in conjunction with the human behaviour recorded in this study, the majority of exposure occurs indoors. However, surveillance must be maintained to detect any shift in behaviour and to monitor exophagic populations of potential secondary vectors. Greater exposure to primary vector bites occurs indoors in the early evening when LLINs are not used. The early biting habit of these vectors was shown to reduce the protective efficacy of LLINs, although the actual estimate of protective efficacy calculated here does not take into account the mass effect on mosquito populations when an entire community uses nets. There are indications that exposure and therefore protective efficacy of nets varies with both an individual’s age and across seasons. A key aspect of man-vector contact is the behaviour of the human local population, and this is not static across the seasons. These results indicate that LLINs may theoretically reduce malaria vector exposure if used correctly, but that other measures are required to protect against early indoor biting. Regular surveillance of both vector behaviour and domestic human-behaviour patterns are needed for the planning of future control interventions in this region.

## Additional file

Additional file 1: Calculation of true bed net protective efficacy. The document details the method used to calculate true bednet protective efficacy.

## Abbreviations

CSP: circumsporozoite protein
ELISA: enzyme-linked immunosorbent assay
EIR: entomologial inoculation rate
GMEP: Global Malaria Eradication Programme
HLC: human landing catches
IRR: incident rate ratio
IRS: indoor residual spraying
ITN: insecticide-treated nets
KEMRI/CDC: Kenya Medical Research Institute/Centers for Disease Control and Prevention in Kisian, Kisumu
LLIN: long lasting insecticidal net
MBR: man biting-rate
P*: true protective efficacy
PCR: polymerase chain reaction
