# Supplementary material for: ‘A bite before bed’: exposure to malaria vectors outside the times of net use in the highlands of western Kenya
Source: Malar J. 2015 Jun 25;14:259. doi: 10.1186/s12936-015-0766-4 (PMC4479228; doi:10.1186/s12936-015-0766-4)
Supplement: Additional file 1: — Calculation of true bed net protective efficacy. The document details the method used to calculate true bednet protective efficacy. [file 12936_2015_766_MOESM1_ESM.docx]

**Additional file 1**

**Calculation of true bednet protective efficacy**

Human exposure to malaria vectors and the true protective efficacy of bednets was calculated using methods adapted from the work described by Geissbuhler *et al.*, (2007) based on the formulae published by Killeen *et al.*, (2006) [1, 2]. These earlier studies calculated the protective efficacy of bednets as a result of reduced exposure to *An. gambiae* bites, incorporating the proportion of the population indoors but not asleep and those indoors and asleep under an LLIN. In the current paper calculations were made separately for *An. funestus* s.l, *An. arabiensis* and *An. gambiae* s.s., as well as for the three vectors combined.

Two calculations were made for each individual using the times individuals entered and exited the house and bed: 1) Where it was assumed that no bednet had been used (B_u_) (Equation 1 and Equation 2), 2) Where it was assumed that a bednet had been used (B_p_) (Equation 3 and Equation 4).

$$B_{u,h}=B_{o,h} \left( \frac{m_{o,h}}{60} \right)+B_{i,h}\left( \frac{m_{i,h}}{60} \right)$$

Equation 1. Mean hourly biting rate experienced by an unprotected (without a net) (u) individual for a specific hour of the night (h). A product of the mean outdoor hourly biting rate that hour (B_o,h_) and the fraction of time spent outdoors during that hour (m_o_=minutes outside, divided by 60) added to the mean hourly biting rate inside (B_i,h_) multiplied by the fraction of time spent indoors (m_i_=minutes inside). Adapted from Geissbuhler *et al. (*2007) and Killeen *et al. (*2006) [1, 2].

$$B_{u}=\sum_{h=1}^{24} B_{u,h}$$

Equation 2. Mean daily biting rate experienced by an unprotected individual, calculated by summing hourly biting rates (B_u,h_) for each hour. The assumption is made that Bu,h is zero for hours before 17:30 in the evening and after 06:30 in the morning both indoors and outdoors. Adapted from Geissbuhler *et al. (*2007) and Killeen *et al. (*2006) [1, 2].

$$B_{p,h}=[B_{o,h}\left( \frac{m_{o,h}}{60} \right)]+{[B}_{i,h}\left( \frac{m_{i,h}-m_{n,h}}{60} \right)]+\left[ B_{i,h}\times\left( \frac{m_{n,h}}{60} \right)\times(1-P) \right]$$

Equation 3. Mean hourly biting rate experienced by an individual protected (p) by a bednet. A product of the mean outdoor hourly biting rate that hour (B_o,h_) and the fraction of time spent outside that hour, added to the mean hourly biting rate inside (B_i,h_) multiplied by the fraction of time spent inside not protected by a net, added to the product of the mean hourly biting rate inside (B_i,h_), the fraction of time spent under the net and 1 minus an estimation of the nets’ protective efficacy (P). Adapted from Geissbuhler *et al. (*2007) and Killeen *et al. (*2006) [1, 2].$\mathbf{m}_{\mathbf{i,h}}$ = MBR indoors per hour, $\mathbf{m}_{\mathbf{o,h}}$ = MBR outdoors per hour, $\mathbf{m}_{\mathbf{n,h}}$ = MBR indoors under net per hour.

$$B_{p}=\sum_{h=1}^{24} B_{p,h}$$

Equation 4. Mean daily biting rate experienced by an individual protected by a net, calculated by summing the hourly biting rates (B_p,h_) for each hour. The assumption is made that the B_p,h_ is zero for the hours before 17:30 in the evening or after 06:30 in the morning. Adapted from Geissbuhler *et al.* (2007) and Killeen *et al. (*2006) [1, 2].

A limitation of this method is the necessary assumption that the protective efficacy of the bednets (P) used in Equation 3 is uniform between houses, and that each individual used an identical model of bednet, and used it correctly. There was a mass distribution of LLINs during this study, but there was evidence of previous multiple LLIN ownership within the recruited households so that those nets being used could vary in age, condition and the insecticide and material used. Two calculations of B_p_ were made for this study. One was based on the theoretical ideal scenario that LLIN were 100% effective at reducing bites (P=1) when used correctly. The other based on the more conservative estimate of a functional protective efficacy of 80% (P=0.8), which had been adopted by previous studies informed by existing evidence from experimental hut trails of ITNs [1, 2].

Therefore, it was possible to calculate an individual’s exposure to vectors, both indoors and outdoors, and the reduction in bites that could theoretically be achieved if a bednet was used (Equation 5 and Equation 6). This permitted a comparison between population age groups and different seasons within the sampling year. The proportion of bites that an individual would experience indoors relative to outdoors ($\pi_{i})$ and vice versa ($\pi_{o})$ could also be calculated for each person (Equation 7 and Equation 8) and, therefore, for each age group and month of sampling (Equation 8 and Equation 9).

$$\lambda_{p}=B_{p}/B_{u}$$

Equation 5. Estimate of relative biting rate (λ) for bednet users, adapted from Geissbuhler *et al.* (2007) and Killeen *et al. (*2006) [1, 2]

$$P*=1-\lambda_{p}$$

Equation 6. Estimate of the true protective bednet efficacy (P*), adapted from Geissbuhler *et al.* (2007) and Killeen *et al. (*2006) [1, 2]

$$B_{iue,h}=B_{i,h}\times\left( \frac{m_{i,h}}{60} \right)$$

$$B_{oe,h}=B_{o,h} \times\left( \frac{m_{o,h}}{60} \right)$$

$$\pi_{\mathrm{iu}}=\sum_{h=1}^{24} \left[ B_{ie,h} \right]/\sum_{h=1}^{24} \left[ B_{oe,h} +B_{ie,h} \right]$$

Equation 7. The proportion of exposure (e) in the form of mosquito bites occurring indoors (π_i_) for an unprotected individual, adapted from Geissbuhler *et al. (*2007) and Killeen *et al. (*2006) [1, 2].

$$\pi_{o}=1-\pi_{i}$$

Equation 8. The proportion of bites occurring outdoors (π_o_) for an unprotected individual. adapted from Geissbuhler *et al. (*2007) and Killeen *et al. (*2006) [1, 2].

$$B_{ipe,h}=B_{i,h}\times\left( \frac{m_{i,h}-m_{n,h}}{60} \right)+\left[ B_{i,h}\times\left( \frac{m_{n,h}}{60} \right)\times(1-P) \right]$$

$$\pi_{\mathrm{ip}}=\sum_{h=1}^{24} \left[ B_{ipe,h} \right]/\sum_{h=1}^{24} \left[ B_{oe,h} +B_{ipe,h} \right]$$

Equation 9.The proportion of bites occurring indoors for an individual protected by a bednet, adapted from Geissbuhler *et al. (*2007) and Killeen *et al. (*2006) [1, 2]

$$\pi_{\mathrm{op}}=1-\pi_{\mathrm{ip}}$$

Equation 10. The proportion of bites occurring outdoors for an individual protected by a bednet. adapted from Geissbuhler *et al. (*2007) and Killeen *et al. (*2006) [1, 2]

References

1. Geissbühler Y, Chaki P, Emidi B, Govella NJ, Shirima R, Mayagaya V, Mtasiwa D, Mshinda H, Fillinger U, Lindsay SW, Kannady K, de Castro MC, Tanner M, Killeen GF: Interdependence of domestic malaria prevention measures and mosquito-human interactions in urban Dar es Salaam, Tanzania. Malar J 2007, 6:126.

2. Killeen GF, Kihonda J, Lyimo E, Oketch FR, Kotas ME, Mathenge E, Schellenberg JA, Lengeler C, Smith TA, Drakeley CJ: Quantifying behavioural interactions between humans and mosquitoes: evaluating the protective efficacy of insecticidal nets against malaria transmission in rural Tanzania. BMC Infect Dis 2006, 6:161.
